# Individual evaluation of fatigue at work to enhance the safety performance in the construction industry: A systematic review

**DOI:** 10.1371/journal.pone.0287892

**Published:** 2024-02-07

**Authors:** Pei Pei Heng, Hanizah Mohd Yusoff, Rozita Hod

**Affiliations:** Department of Public Health Medicine, Faculty of Medicine, Universiti Kebangsaan Malaysia, Cheras, Wilayah Persekutuan Kuala Lumpur, Malaysia; Federal University of Pernambuco: Universidade Federal de Pernambuco, BRAZIL

## Abstract

The construction industry is recognized as one of the most hazardous industries globally due to the dynamic on site activities and labour-intensive characteristics. The construction tasks are physically and cognitively demanding therefore the construction workers are prone to work fatigue which compromises safety performance. The evaluation of fit for duty, or fitness for work (FFW) aims to determine if workers are at risk of adverse impacts of ill-health, injury or accidents. This systematic review aimed to critically summarize up-to-date measures and evaluation tools that were employed to monitor work fitness or fatigue specifically among construction workers. Adhering with the PRISMA protocol, three databases were searched from the inception to 2022, with a total combination of 37 keywords, concluding to the selection of 20 relevant articles. The Mixed Method Appraisal Tool (MMAT) was used as the guide for the study appraisal. A total of 20 articles were reviewed, published from 2008–2022. Majority of the studies employed experimental design. The review identified the subjective evaluation scales and objective measurement tool. The subjective self-response questionnaires can be categorized into single dimension or multidimension covering both physical and mental fitness; whereas the objective measurement tool can be categorized into physiological metrics, physical and cognitive performance measure. The available scientific evidence has raised the relevant issues for on-site practicality and potentially guide the formulation of evidence-based guidelines for the FFW assessment in the construction industry.

## 1. Introduction

The Fitness for Work (FFW), or “fit for duty” evaluation is a comprehensive functional assessment of a worker’s capacity to perform work tasks without jeopardising their own or others’ occupational health and safety [1]. The assessment aims to identify if workers are not fit for work due to the risk of adverse impacts of ill-health, injury, accidents or fatality [2]. The construction industry represents high risk for workers’ safety compared to other industries, due to its dynamics on site activities and complex settings; with labor-intensive characteristics [3, 4]. The scaffolders, steel fixers, form workers, electrician plumbers, concreters and other manual handling labourer are often categorized as workers with physically demanding and exhausting job that are prone to work fatigue [5, 6]. The overextended fatigue is highly associated with human error [7]. Construction tasks requires sequential procedural steps and an optimum level of alertness [8]. Certain functional fitness requirements for performing construction jobs, such as postural stability, balancing, muscle strength and endurance, and cognitive impairment, are difficult to be noticed, identified, measured, and reported [9]. A reduced physical capabilities and lapse in memory may therefore compromise the task performance, turning the routine task into hazardous task.

Globally, as high as 6,000 death among workers due to construction accidents were estimated annually [10]; with a major proportion (80%) reported due to individual attribute [11–13]. Occupational accidents result in devastating socioeconomic consequences because, in addition to causing physical and mental disability, fatal accidents have significant personal, societal, and financial costs [14]. Conventionally, passive safety counter measures have been undertaken in the prevention of construction accidents, including the on-site precautionary measures of Personal Fall Arrest Systems, guardrails, safety nets, harness, workers’ training in accordance with safety regulations, and task redesign. However. the overemphasize of technical and managerial factors rather than individual attributes such as fatigue, did not improve the construction accidents statistics substantially [15]. Counter measures to tackle the individual attributes such as work fatigue should therefore be explored and promoted. When workers are in the state of physical and cognitive degradation, their information processing ability is significantly reduced followed by a cascade of effects, such as diminished attentiveness [16], decrease reaction time in response to stimuli [17, 18]. This compromises decision-making abilities, as a result, triggers unsafe behavior [19], disrupts the safety of the workplace hence resulting in errors, risky conduct, injury, and mortality [16]. Fang et al. [20] while demonstrating the relationship between work fatigue level and safety performance, reported that workers were more likely to involve in errors and accidents when they were less fit for performance.

Fatigue at work evaluation should be tailored to the functional capacity requirements and risks of the job. In other words, the scope of assessment is customized and varies between occupation and job task [26, 31]. It was frequently evaluated among certain occupational groups including the army [21], healthcare providers [22], air crews [23], drivers [24] and factory workers [25]. On the other hand, Serra et al. (2007) in a critical review reported majority of the functional fitness assessment tools applied to individual worker were laboratory diagnostic tests which are invasive and time consuming [26]. Some of the examples of these assessment tools are urine drug screening test among drivers [27]; obesity and cardiovascular risk screening among the firefighters [28]; lung function assessment among miners [29], muscular strength, core stability, flexibility and balance among the astronaut’s crops [30].

The aggressive pace and rhythm at construction site can be super sensitive to the movement deceleration, in comparison to other works in relatively stable environment such as manufacturing and transportation industry. The FFW assessment must be tailored to the job function and work scenario [31, 32]. Those assessment tools employed in other industries might not be appropriately applied to the construction workers. Therefore, present critical review aims to systematically summarize various parameters used to measure physiological and psychological changes related to fitness and fatigue among construction workers and the potential evaluation tools that can be used to monitor work fitness capacity. Additionally, the type, scope and potential challenge of the worker-oriented measures will be discussed with the provision of future research directions in order to achieve the International Labour Organization’s aim of zero harm in the occupational setting.

## 2. Materials and methods

### 2.1. Formulation of research question

This review employed a systematic approach to an extensive search on relevant articles, followed by critical appraisal of the work fitness measures among the construction workers, as well as the encapsulation the type, scope and challenges of each FFW assessment tool. The relevant research question was formulated based on the three major concept in the PICO approach, namely: Population or Problem (construction workers), Interest (assessment tool or instrument), and Context/Outcome (fit for duty/fatigue) [33], which have guided the synthesis of the main research question ‘What are the potential assessment tools used to evaluate fitness for duty and monitor work fitness capacity among the construction workers in order to minimize the risk of work-related ill-health and injury?

### 2.2. Search strategy

**Fig 1:**
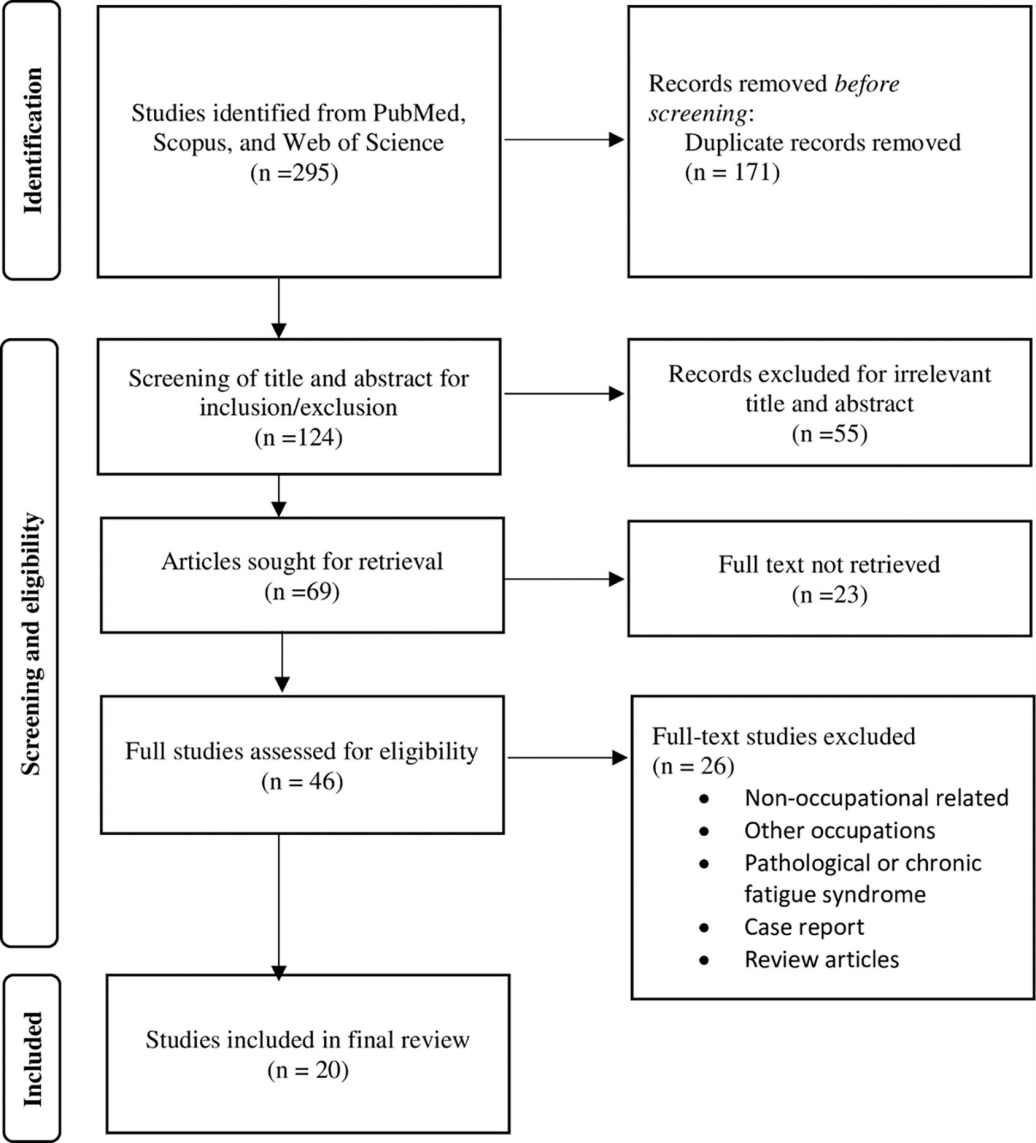
PRISMA 2020 flow diagram for scoping reviews which included searches of databases, registers and other sources.

The literature search was conducted in December 2022. Three databases were included, namely Scopus, PubMed and Web of Science. The 3 groups of keywords used for the searching of relevant articles are shown in Table 1. The combination of all groups of keywords using “AND” has produced a total of 295 papers, from the three databases. (Fig 1).

**Table 1:** Search terms and keywords.

	PubMed	Scopus	Web of Science
Construction worker* OR Construction industry OR. Construction trade OR Construction sector OR Industrial Construction OR Construction OR building OR building workers	134,677	143,529	576,715
Fitness OR Work Fitness OR Fitness for Work OR Functional Fitness OR Fit to Work OR Fit for duty OR Fitness to Work OR Fatigue OR Exertion OR Tiredness OR Physical Lassitude OR physical effort OR muscle fatigue OR Physical fatigue OR Cognitive Fatigue OR Cognitive Exhaustion OR Cognitive Lassitude OR Mental Fatigue OR Mental Exhaustion OR Mental Lassitude	129,528	138,673	325,433
Assessment OR Assess OR Examination OR Evaluation OR Estimation OR Valuation OR Analysis OR Assessment tool OR Tool OR Instrument	172,653	177,543	425,436
Combined (intersection of articles by the combination of 3 groups of explicit keywords)	72	69	154
Total after duplication removed		124	

### 2.3. Selection criteria

Articles were selected based on specific inclusion criteria of: (1) original research; (2) written in English; (3) observational and experimental study relevant to the research question. The article was excluded if the outcomes related to fitness for work evaluation were not reported and not occupational related. Other exclusion criteria are: animals’ studies, review articles, case reports, newsletters, commentaries, conference proceedings, and grey literature.

### 2.4. Screening for eligibility and data extraction

All online search results (n = 295) were exported into EndNote 20.1, and duplicates were removed (n = 171). For the remaining 124 articles, abstract was read if uncertainties raised in the title. Two reviewers completed the screening of titles and abstracts and 55 non-relevant articles were removed, while the remaining articles were retrieved of full text (n = 69). There were 23 articles cannot be retrieved, leaving 46 articles for full-text assessment and eligibility screening. The relevant full-text articles were reviewed by the two independent reviewers. Any disputes or discrepancy between the two reviewers were resolved by the third reviewer. At the screening phase, all articles were compared against a pre-determined set of inclusion and exclusion criteria. The articles were considered eligible only if the study population was specific among the construction industry; and the outcome variable focus on the objective or subjective or combined assessment tool in order to evaluate “fitness for duty” in the perspective of physical or cognitive capacity. As a result, a total of 26 articles were excluded as the study were not occupational related, focused on occupations other than construction industry, irrelevant measure of pathological fatigue or chronic fatigue syndrome. Subsequently, the 20 remaining articles proceeded for data extraction including authors/year, country, population, study design, sample size, assessment tool (subjective or objective or combined), scope of evaluation (physical or cognitive fitness), results, strength or potential challenges.

### 2.5. Quality appraisal

Quality appraisal was conducted using the Mixed Method Appraisal Tool (MMAT) [34]. The MMAT is a critical appraisal tool developed to appraise methodology quality of five types of studies, namely qualitative studies, randomized control trials, non-randomized studies, quantitative descriptive study, and mixed methods study in a review article. The assessment based on five main criteria: sampling strategy relevant to address the research question; sample representative of the target population; measurements appropriateness; the risk of non-response and appropriateness of statistical analysis. The scores of qualities were reported as an overall score (5*****/100% quality criteria met; 4****/80% quality criteria met; 3***/60% quality criteria met; 2**/40% quality criteria met; 1*/20% quality criteria met) (S1 Appendix).

## 3. Results

There was a total of 20 articles reviewed in this study. The descriptive summary on characteristics of all included articles, including year of publication, study location and study design are tabulated in Table 2. The review articles were published from 2008 to 2022; one in 2018, two in 2009, two in 2014, three in 2015, three in 2017, four in 2018, two in 2020, two in 2021, one in 2022. Most of the studies were conducted in Asia: Taiwan (n = 4) [35–38], Hong Kong (n = 3) [39–41], India (n = 2) [42, 43], Korea (n = 1) [44], China (n = 1) [20]; followed by United States(n = 3) [32, 45, 46], Brazil (n = 2) [7, 47], and one article each for New England [8], Poland [48], Iran [49] and Chile [50]. Two-fifth of the studies (n = 8) employed experimental design in which three used simulated construction tasks. Another two-fifth (n = 8) employed cross-sectional design, while three employed longitudinal time series design and only one with case control design. The objectives, study population, details of assessment tool and research findings of all studies were summarized in Table 3.

**Table 2:** Descriptive characteristics of all included studies.

Study location	Source
Taiwan	Chang et al. (2009); Hsu et al. (2008); Li et al. (2009); Tsai (2017)
Hong Kong	Anwer et al. (2020); Umer et al. (2018); Wong et al. (2014)
United States	Aryal et al. (2017); Techera et al. (2017); Zhang et al. (2015)
India	Das (2014); Mohapatra et al. (2022)
Brazil	Correia et al. (2018); Galati et al. (2020)
Korea	Lee et al. (2021)
China	Fang et al. (2015)
New England	Zhang et al. (2015)
Poland	Cyma et al. (2018)
Iran	Khavanin et al. (2018)
Chile	Ferrada et al. (2021)
**Study design**	
Experimental	Li et al. (2009); Chang et al.(2009); Das (2014); Wong et al. (2014); Fang et al.(2015);Aryal et al. (2017); Tsai (2017); Anwer et al. (2020);
Cross-sectional	Zhang et al. (2015a); Zhang et al. (2015b);Techera et al. (2017);Khavanin et al (2018);Galati et al. (2020);Ferrada et al. (2021); Lee et al.(2021); Mohapatra et al.(2022)
Longitudinal time series	Hsu et al.(2008);Correia et al. (2018); Umer et al. (2018)
Case control	Cyma et al. (2018)

**Table 3:** Characteristics and findings of the included studies.

No	Source	Study design	Objective	Study population	The potential work fatigue evaluation tool	Instrument, test protocol, content and measurement scale.	Findings
1	Hsu et al. 2008 Taiwan [35]	Longitudinal time series design (pre and post shift)	To investigate the effect of elevation, change on the prevalence rates of subjective fatigue symptoms and physiological responses	80 construction workers working at height	1. calf circumference, blood pressure, heart rate, critical flicker fusion (CFF) and muscle strength for pinch, grip and back. 2. Subjective fatigue symptoms RCIF scale designed by the Research Committee on Industrial Fatigue of Japan Society for Occupational Health	Wrist blood pressure meter (Terumo, Model ES-P2000, Japan) was used to measure worker heart rate and blood pressure. Strength test measures by Takei dynamometer (Japan). CFF was measured with Takei digital flicker (Model 502, Japan) with a frequency range of 20–60 cycles/s. 30 items, 3 domains (cover physical and mental dimension): ‘‘drowsiness and dullness”, ‘‘difficulty in concentration”, and the ‘‘the projection of physical disintegration”	The post-shift prevalence rates of subjective fatigue symptoms and heart rate among high-rise building construction workers were found to increase at successively increasing elevations. Strength test showed strength after work was greater than that before work, indicated psychological factors may be involved. Elevation change was also shown to affect workers’ visual sensitivity
2	Li et al. 2009 Taiwan [36]	Experimental	To investigate the physiological and perceptual responses in male Chinese workers performing combined manual materials handling tasks	8 construction workers handling box lifting task	Oxygen uptake and heart rate. Rating of perceived exertion (RPE) for whole body measured during the lifting task using Borg scale 6–20	Actual energy expenditure of the box handling for an hour was calculated from the oxygen uptake measured, whereas the predicted energy expenditure was estimated using the valid regression equations	Both task frequency and lifting and lowering heights influence oxygen uptake, heart rate, and the RPE. The RPE during the task frequency of twice per minute was higher than that of once per minute. Predictive equations used in this study are acceptable in estimating the physiological cost of Chinese construction workers performing similar manual handling tasks as in this study.
3	Chang et al. 2009 Taiwan [37]	Experimental (One group pre-post design)	To investigate whether work fatigue and physiological symptoms experienced by high-elevation construction workers affected by the occupations	302 high-rise building construction workers of scaffolders, steel fixers, form workers, electrician-plumbers, concreters and miscellaneous workers.	1. Subjective Fatigue symptoms Questionnaire developed by the Research Committee on Industrial Fatigue, Japan 1969. 2. Physiological measurement 3. Physical performance measure	30 items: 3 domains on drowsiness and dullness, difficulty in concentration, projection of physical impairment (Dichotomous: Yes/No) Heart rate Calf circumference strength tests of pinch, grip and back	Variation of average heart rate and strength test was highest among scaffolders (physical demanding task) and lowest among concreters (general type worker). High elevation workers have more complaints of subjective fatigue symptoms, with the potentially greater complaint of emotional stress and physiological strain.
4	Das 2014 India [42]	Experimental (pre and post shift with control group)	to determine the physiological stress among brick field workers compared to control workers.	220 construction workers handling brisk field works vs 130 control subjects	Heart rate and blood pressure	Manual measurement prior to work and post shift	The participants HR rose to > 100 bpm. The average HR of brick field workers was 148.6 bpm after the construction tasks. Brick field workers had severe physiological stress as indicated by increased HR.
5	Wong et al. 2014 Hong Kong [41]	Experimental field study	To quantify the respective physical workloads of bar bending and fixing; and (2) compare the physiological and perceptual responses between bar benders and bar fixers.	39 rebar workers	energy expenditure, minute ventilation, heart rate, and oxygen consumption Borg CR10 Scale to evaluate physical load	Oxygen consumption was expressed as (ml/min) d relative to participant’s body weight (ml/min/kg). Maximum heart rate was calculated using the age-predicted equation: Maximum heart rate = 208–0.7*age	Bar fixing task induced significantly higher heart rate than bar bending task. Heart rate can be used to assess physical fatigue during rebar working.
6	Fang et al. 2015 China [20]	Experimental design to simulate the actual construction work of manual handling	to study the effect of fatigue on construction workers’ safety performance	20 rebar workers	The Fatigue Assessment Scale for Construction Workers (FASCW)	5-point Likert scale 10 items, 2 dimensions: lethargy, bodily ailment.	Fatigue level of 20 was a critical point from where the effect of fatigue began to emerge.When a worker’s fatigue level exceeded 20, there was a linear relationship between fatigue levels and error rates mainly failure of hazard perception. As fatigue accumulated, its impact on the worker’s capacity of motor control became significant.
7	Zhang et al. 2015 New England [8]	Cross-sectional		606 construction workers	Self-reported survey	Feeling of fatigue with one survey question: ‘In the past 3 months, how often did you feel very tired or exhausted?’ (Never, some days, most days or every day) Physical function assessed through questions about difficulties with nine daily functional activities (no difficulty/with difficulty). 1. Walk a quarter of a mile—about 3 city blocks? 2. Walk up 10 steps without resting? 3. Stand or be on your feet for about 2 hours? 4. Sit for about 2 hours? 5. Stoop, bend, or kneel? 6. Reach up over your head? 7. Use your fingers to grasp or handle small objects? 8. Lift or carry something as heavy as 10 pounds such as a full bag of groceries? 9. Push or pull large objects as heavy as 10 pounds such as a living room chair? Cognitive function with the question: ‘Do you have difficulty remembering or concentrating? (no difficulty/with difficulty)	There was an association between reported fatigue and experiencing difficulties with physical and cognitive functions in construction workers. Almost half of the respondents reported being ‘tired some days’ in the past 3 months and 1 in 10 reported ‘tired most days or every day’. Compared with those feeling ‘never tired’, workers who felt ‘tired some days’ were significantly more likely to report difficulty with physical and cognitive function.
8	Zhang et al. 2015 Unites States [32]	Cross sectional	To assess the reliability, validity and sensitivity of the newly constructed fatigue scale of FASCW	144 construction workers	The Fatigue Assessment Scale for Construction Workers (FASCW)	5-point Likert scale 10 items, 2 dimensions: lethargy, bodily ailment.	The 10-item FASCW with good reliability and validity is an effective tool for assessing the severity of fatigue among construction workers.
9	Aryal et al, 2017 United States [45]	Experimental woth simulated material handling construction task	For real time monitoring of physical fatigue in construction workers using wearable sensors.	12 construction workers	heart rate, thermoregulation and electrical brain activity Borg scale 6–20	Heart rate monitor, infrared temperature sensors and an EEG sensor were used to monitor physiological response during simulated task.	Heart rate and skin temperature sensor signals can be used to predict the level of physical fatigue using wearable sensor.
10	Techera et al. 2017 United States [46]	Cross-sectional	To determine a set of objective variables can predict variability in construction worker fatigue.	252 US construction workers	1. Swedish Occupational Fatigue Inventory (SOFI) 2. 5 minutes personal computer version of the Psychomotor Vigilance Test (PC-PVT)	7-point Likert-scale 20 items and 5 dimensional subscales: lack of energy, physical exertion, physical discomfort, lack of motivation, and sleepiness. higher scores indicate a greater severity of momentary fatigue in the here and now. The participant observes a black screen, a stimulus appears in the form of red four-digit millisecond counter that stops the count once the participant clicks the mouse. The counter displays the reaction time (RT) of the individual for 500 ms and then disappears. This sequence repeats at random intervals. between 2 and 10 seconds	Good relationship with the supervisor and an increase in the amount of sleep obtained in the past 24h would contribute to a lower RT or SOFI score. The longer the recovery period, the faster the RT or lower SOFI score they would obtain. The objective and subjective fatigue measurement tools identify different dimensions of fatigue and both should be considered to measure fatigue
11	Tsai 2017 Taiwan [38]	Experimental	To apply physiological status monitoring in improving construction safety management	20 general construction workers	Physiological Status Monitoring (PSM)	Brain wave rhythms and Heart Rate Variability (HRV) detection using Photo Plethysmography-based wearable device	Physiological status monitoring of workers using HRV identified more fatigue risk than manual inspection. Assessment of HRV is a useful approach to evaluate real-time fatigue during construction tasks. The proposed approach could analyze fatigue levels and help identifying risks of fatigue, in order to notify the fatigued workers as well as transfers relevant statistics to construction managers. The managers, therefore, are able to supervise their workers in real time.
12	Correia et al. 2018 Brazil [7]	Longitudinal time series design (at 0730, 1130 and 1730 hour)	to evaluate and investigate factors that affect the fatigue of construction workers	15 construction workers	The Fatigue Assessment Scale for Construction Workers (FASCW) Heart rate	5-point Likert scale 10 items, 2 dimensions: lethargy, bodily ailment.	FASCW allowed to evaluate both physical and mental fatigue. Level of fatigue and heart rate increased as the working day progressed. Among workers who exceeded critical point of 20, the prevalence of physical fatigue with leg and joint pain symptoms increases. Group of workers aged 30–39 reported highest average fatigue.
13	Cyma et al. 2018 Poland [48]	Case-control	to analyze the level of postural stability and physical activity of construction workers working at height compared to office workers	17 workers at height compared to 17 office workers	1. Baecke questionnaire 2. One-leg standing test with eyes open and closed	To assess physical activity at work, sports activity, and leisure (3 levels of intensity of work activity, 3 levels of sports intensity, and 5 levels of frequency of performed activities) Subject passes the test with eyes open after 45 s and later, with eyes closed after 15 s	The at-height workers group had a higher rate of average physical activity at work. The groups differed in terms of postural stability in favor of at-height workers. Postural stability is rather affected by exposure to distress conditions among construction workers working at height
14	Khavanin et al. 2018 Iran [49]	Cross-sectional	to investigate the physical and mental fitness of telecommunication tower climbers as well as their job stress.	60 employees of a contracting company which worked in the field of telecommunication tower installation	1. Work Ability Index (WAI) Health and Safety Executive (HSE) Stress	7-point Likert scale 7 items: current ability, work ability in relation to physical and mental demands of the job, reported diagnosed diseases, estimated impairment due to health status, sick leave over the last 12 months, self-prognosis of work ability in the 2 years to come and mental resources of the individual.5 point-Likert scale. 35 questions with 7 criteria including demand (8 items), control (6 items), managerial support (5 items), peer support (4 questions items), relationships (4 items), role (5 items) and change (3 items)	There was a significant relation between WAI and educational level, job tenure, hours of sleep per day, regular exercise, and second job. Among the dimensions of work related stress, control and changes were significant predictors of the WAI score. To improve the worker’s work ability, intervention programs should focus on promoting level of job control, sleep quality and exercise.
15	Umer et al. 2018 Hong Kong [39]	Longitudinal time series design	To develop a static balance monitoring tool for proactive tracking of construction workers on-site using a wearable inertial measurement unit (WIMU) and a smartphone.	13 construction workers	Static balance test	WIMUs was used to detect task/fatigue-induced changes in static balance during a 20-second static balance test.	WIMUs could detect the post-task subtle changes in static balance with reference to the findings of a force-plate. Mobile phone application allowed managers/foremen for onsite balance monitoring of the construction workers using the 20-second test and assist early identification of fall prone workers, plan mitigation schemes before a fall accident happens in the construction industry.
16	Anwer et al. 2020 Hong Kong [40]	Experimental design to simulate the actual construction work	to quantify real-time physical fatigue using during a simulated construction task	25 construction workers	1. wearable cardiorespiratory and thermoregulatory sensors (EQ02 Life Monitoring System) 2. Borg-20 scale for the perceived fatigue at“maximal exertion” of effort	Heart rate, breathing rate, local skin temperature, and electrodermal activity at the wrist were measured by wearable sensors and the perceived physical fatigue was assessed at baseline, 15 min, and 30 minutes during fatigue task.	Cardiorespiratory parameters and local skin temperature were good surrogates for measuring physical fatigue. There were significant increases in the heart rate , breathing rate, local skin Temperature, electrodermal activity and subjective physical fatigue at the end of the simulated construction task. Heart rate and breathing rate at 15 and 30 min were significantly correlated with the corresponding subjective Borg scores while local skin temperature at 30 min was significantly correlated with the corresponding Borg scores.
17	Galati et al. 2020 Brazil [47]	Cross-sectional	To identify the factors associated with occupational accidents for construction workers, in particular their work ability index (WAI) and the quality of life at work (QLW/SF-36).	114 construction workers	Work Ability Index (WAI)	Self-reported current ability compared to the lifetime best. 7-point Likert scale 7 items: current ability, work ability in relation to physical and mental demands of the job, reported diagnosed diseases, estimated impairment due to health status, sick leave over the last 12 months, self-prognosis of work ability in the 2 years to come and mental resources of the individual.	Work ability index (WAI) was associated with the occurrence of work accidents. The sample of workers presented a young age profile with 95% of the individuals under the age of 45 years, low education level, good disposition to work with only 5% with low WAI and average score of quality of life around 72%
18	Ferrada et al. 2021 Chile [50]	Cross sectional	To understand the association between sleep duration and fatigue among construction workers and to propose strategies to mitigate them in the reduction of construction accidents.	154 construction workers	1. Pittsburgh Sleep Quality Index (PSQI). 2. personal computer version of the Psychomotor Vigilance Test (PC-PVT)	self-report questionnaire that assesses sleep quality over a 1-month time interval. Consists of 19 items, creating 7 components that produce one global score ranging from 0 to 21 with the higher score indicating worse sleep quality. Reaction time (RT) measures alertness and vigilance.	Less than a quarter of the sample presented good sleep quality (Pittsburgh <5) and two-thirds with sleep <7 h. PC-PVT test is the objective means of evaluating fatigue. Better performance in the test was observed in the group that reported sleeping between 5 h and 7 h per day on average
19	Lee et al. 2021 Korea [44]	Cross sectional	To evaluate the psychometric properties of a Korean version of the SOFI among construction workers.	193 construction workers	Swedish Occupational Fatigue Inventory (SOFI)	7-point Likert-scale 20 items and 5 dimensional subscales: lack of energy, physical exertion, physical discomfort, lack of motivation, and sleepiness. higher scores indicate a greater severity of momentary fatigue in the here and now.	Korean version of the SOFI is a reliable and valid instrument to evaluate momentary work-related fatigue among construction workers with satisfactory internal consistency reliability, item–subscale reliability, and test–retest reliability Positive concurrent validity was reported with good correlation with the Multidimensional Fatigue Scale (MFS) and Subjective Symptoms of a Fatigue test (SSF)
20	Mohapatra et al. 2022 India [43]	Cross sectional	to develop a lifting capacity prediction model for construction workers based on muscle strength and endurance.	65 construction workers with manual handling	Physical performance assessment of core strength and endurance, grip strength, and lower limb flexibility	Lifting capacity (PILE) Bilateral handgrip strength measured in kg Sit & reach test measured in cm, while Prone plank test, Trunk flexor endurance test, Trunk extensor endurance test, Trunk lateral flexor endurance test were measured in seconds where the participants could maintain the core muscular strength and endurance.	The age, BMI, grip strength, flexibility, prone plank, and trunk lateral flexor endurance tests have significantly influenced lifting capacity. Regression model was developed: Lifting capacity in kg = 3.177 − 0.228(age) + 0.868(BMI) + 0.193(grip strength) +0.270(flexibility + 0.204(prone plank time) + 0.165(trunk lateral flexor endurance time) The model would help in easy estimation of lifting capacity among construction workers, which could be even administered with minimal skills by site supervisors or managers, and help in the decision-making during pre-placement or return to work evaluations,

The subsequent systematic analysis identified two main types of fatigue assessment tool, namely the subjective scale and objective measurement. The subjective evaluation tool included self-response or self-administered survey, which was further categorized based on the dimension they measured, namely physical fatigue; or the combination of multidimensional fatigue comprised of physical and mental domains. The Borg scale [36, 40, 41, 45] for the perceived fatigue based on efforts for exertion and the Baecke questionnaire [48] were used to evaluate solely on physical fatigue. On the other hand, validated questionnaire like Work Ability Index (WBI) [47, 49], Subjective fatigue symptoms RCIF scale [35, 37], Fatigue Assessment Scale for Construction Workers (FASCW) [7, 20, 37], Self-reported physical fatigue, physical and cognitive function and Swedish Occupational Fatigue Inventory (SOFI) [44, 46] were able to evaluate both physical and mental fatigue. For the objective measurement tool, it was further categorized based on the performance measure that the tool is able to assess, namely (i) physiological metrics of calf circumference [35, 37], blood pressure [35, 42], heart rate [7, 35–38, 40, 42, 45], oxygen consumption [36, 41], skin thermoregulation [40, 45] and electrical brain activity [38, 45, 46],(ii) physical performance measure of stability test of one leg standing test with eyes closed and opened [48], static balance test [39], core strength and endurance [43], lower limb flexibility [43], muscle strength test (pinch, grip, back) [35, 37, 43]; and (iii) cognitive performance measure of personal computer version of the Psychomotor Vigilance Test (PC-PVT) [46, 50] and critical flicker fusion [35]. The type, scope and challenges of the evaluation tool were illustrated in Table 4.

**Table 4:** The types, scopes and challenges of the work fitness assessment tool.

Type of evaluation tool	Scope of assessment	Parameters/ Evaluation tool	Functionality and utility	Strength/ challenges	Sources
Subjective	**Physical fatigue**	Borg scale (RPE)	Assessment of a person’s perception of their effort and exertion, breathlessness, and fatigue during physical work. It represents an estimate of heart rate by multiplying the score by 10. For example, if the rate for light jog is 13, the heart rate is likely around 130.	Insufficient to capture the whole range of perceptual sensations that worker experience while being physically active.	[36, 40, 41, 45]
		Baecke questionnaire	Evaluation on habitual physical activity on household activities, sports, and leisure time activities, over a time period of one year. Activities are scored on a scale of 1–5 with the total scored from 3–15. A score of 5 indicates the most activity and 1 indicates the least activity for each index.	Subjected to recall or reporting bias due to the long recall period. It does not address cognitive dimension	[48]
	**Physical and mental fatigue**	Work Ability Index (WBI)	It is an instrument commonly used in clinical occupational health and research to assess work ability during health examinations and workplace surveys. The index is determined on the basis of the answers to a series of questions which take into consideration the demands of work, the worker’s health status and resources.	Provide a variety of dimensions on worker’s self-perceived working capacity in relation to demand, health status and mental capacity. However, it is subjected to bias and manipulation of outcome.	[47, 49]
		Subjective fatigue symptoms RCIF scale	The scale was designed by the Research Committee on Industrial Fatigue of Japan Society for Occupational Health to rate the fatigue level by repeated administration of survey: just before work, just after work and just before retiring to bed, with the assumption that the early shift and late shift worker will have more intense fatigue complaint.	Evaluate multidimensional fatigue symptoms however each item only based on the dichotomous (Yes/No) response may induce survey bias and may capture what a respondent really thinks	[35, 37]
		The Fatigue Assessment Scale for Construction Workers (FASCW)	It was developed with the goal of creating a survey instrument capable of assessing self-reported mental and physical fatigue specifically among the commercial construction workers, taking into consideration the nature of task in the construction industry.	Consisted of only 10-items which is ease to administer among the blue-collar workers in construction site with lower literacy level.	[7, 20, 37]
		Swedish Occupational Fatigue Inventory (SOFI)	The scale was designed to identify different dimensions of work-related perceived fatigue in various occupational groups. The five-factor structure of the SOFI (lack of energy, physical exertion, physical discomfort, lack of motivation and sleepiness) was reported related to changes in the physiological parameters associated with fatigue, such as EEG and EMG measurements.	Able to capture the momentary symptom therefore are useful to examine acute or immediate fatigue in workers’ daily working lives.	[44, 46]
		Self-reported physical fatigue, physical and cognitive function	It is easy to administer, simple and quick in the screening for self-reported physical and mental fatigue based on dichrotomous response (Yes/ No),	The use of single item measure for each domain might be unable to capture the construct (low content validity), have fewer points of discrimination (sensitivity), and lack a measure of internal-consistency reliability (reliability)	[8]
Objective	**Physiological measurement**	Calf circumference	It provides information about normal muscle mass and reflects an increase or decrease in muscle mass following physical activity. Calf circumference is a representative anthropometric index that may be useful for screening sarcopenia. In the cases of fatigue evaluation, an increase calf circumference following heavy lower limb activity mst be in line with other parameters of fatigue.	Might require the worker to stop and dedicate time to the assessment.	[35, 37]
		Blood pressure	Based on the physiological basis of fatigue, fatigued individuals had larger blood pressure increases than rested individuals	Might require the worker to stop and dedicate time to the assessment.	[35, 42]
		Heart rate	Based on the physiological basis of fatigue, fatigued individuals had larger heart rate increases than rested individuals. The physiological index of heart rate has also been recognized as the reliable measure to monitor physical degradation.	The continuous monitoring requires the participant to carry a wearable device, which can be intrusive and affect task performance	[7, 35–38, 40, 42–45]
		Oxygen consumption	Oxygen consumption (V02) rises rapidly at the onset of heavy exertion, with an accompanying increase in carbon dioxide production and a small increase in blood lactate. The average oxygen uptake for the measured construction activities was 0.82 L⋅min^−1^ (±0.22 L⋅min^−1^).	The continuous monitoring requires the participant to carry a wearable device, which can be intrusive and affect task performance	[36, 41]
		Skin thermoregulation	Skin temperature probes or sensors are used for continuous skin temperature monitoring. The skin will be used as an indicator of body temperature, which will increase with increasing physical exertion	The continuous monitoring requires the participant to carry a wearable device, which can be intrusive and affect task performance	[40, 45]
		Electrical brain activity	Construction workers frequently experience mental fatigue owing to the high cognitive load of their tasks in a dynamic, complex environment. Work fatigue can be identified by monitoring several brain waves. Alpha waves reflects a state of relaxed wakefulness which decrease with concentration, stimulation, or visual fixation, in a state where the worker is fatigued enough to fall asleep. On the other hand, Beta waves are increased while alert and decreases during drowsiness.	Might require the worker to stop and dedicate time to the assessment.	[38, 45, 46]
	**Physical performance measure**	Stability test of one leg standing test with eyes closed and open	It is used to assess static postural and balance control, clinically to monitor neurological and musculoskeletal conditions. The ability to control anticipatory postural adjustments prior to lifting one leg while standing in unsupported equilibrium represents a complex motor task that is significantly impaired by neurological or lower extremities pathology.	Need full understanding and technical cooperation from the worker. Any deviation from the procedure might result in false negative finding.	[48]
		Static balance test	The test consists of five posture-holding tasks (sitting, stride standing, close standing, one-foot standing on the unparalysed leg, and one-foot standing on the paralyzed leg). Four grades, 1–4, are used to judge the ability of patients to hold these postures, based on the individual’s ability to hold four progressively more challenging positions.	Require artificial intelligence implementation in real-life occupational settings and is costly	[39]
		Core strength and endurance test	Strength of core muscles are among the important physical performance measures among the construction workers as they predict abdominal muscle functionality while the fall arrest system like harness and lanyard are worn. muscular fitness The muscular assessment consists of muscular-endurance tests, which assess the ability to resist fatigue; and muscular-strength tests, which assess the maximum amount force an individual can produce in a specified number of repetitions.	Need full understanding and technical cooperation from the worker. Any deviation from the procedure might result in false negative finding.	[43]
		Lower limb flexibility	The Sit and Reach Test is a linear flexibility tests which helps to measure the extensibility of the hamstrings and lower back. Better flexibility indicates a better physical performance and more resistant against physical fatigue.	Need full understanding and technical cooperation from the worker. Any deviation from the procedure might result in false negative finding.	[43]
		Muscle strength test (pinch, grip, back)	Grip strength is a measure of muscular strength or the maximum force/tension generated by forearm muscles. It can be used as a screening tool for the measurement of upper body strength and overall strength. It is most useful when multiple measurements are taken over time to track performance.	Need full cooperation from the worker. Any deviation from the procedure will impair the result	[35, 37, 43]
	**Cognitive performance measure**	Personal computer version of the Psychomotor Vigilance Test (PC-PVT)	It objectively assesses fatigue-related changes in alertness associated with sleep loss, extended wakefulness, circadian misalignment, and time on task.	Require artificial intelligence implementation in real-life occupational settings and is costly	[46, 50]
		Critical flicker fusion (CFF)	It is a measure of a visual system’s ability to resolve rapid stimulus change, and is defined as the maximum temporal frequency at which a light can flicker before being perceived as continuous. CFF is useful for assessing the temporal characteristics of the visual system, in order to measure visual fatigue. It is measured as a frequency and expressed in hertz (Hz).	Require artificial intelligence implementation in real-life occupational settings and is costly	[35]

## 4. Discussion

The term “Fit to Work” correlates with work performance. It refers to the physical and mental wellbeing of the workers and their ability to fit well with the job task especially the high-risk work. Unfit for work has been recognized as a consequence of fatigue which can trigger unsafe behavior [51], lead to work error therefore leaving an impact on the safety and increase the likelihood of occupational accidents [19]. The scientific literatures have categorized causes of construction accidents into technical factors, environmental factors, human factors and organizational factors [52]. The technical errors arisen from deficiencies in the plant, equipment, tools or materials handling system such as insecure structure design. Human factors have been highlighted as the main culprit leading to construction accidents which is strongly associated with the individual fitness for work level that could be influenced by fatigue [53]. Human factor analysis further revealed that workers’ unsafe behaviour, violation of the safety rules. experience, PPE practices; are among the attributes that were caused by work fatigue. Fatigue is identified as an influencing factor for the applied capability task demands mismatch which may result in work error. In other words, fatigue may reduce the overall capability, so as to increase the probability of errors term [20].

Construction workers have high level of physical and cognitive demand as a result of an overextended work activity, therefore are prone to fatigue and safety performance degradation [8]. The work fitness assessment can be a huge challenge. Relevant indicators like postural stability, balancing, muscular fatigue, cognitive degradation is typically difficult to be self-recognized [9]. The unfit state is mostly underreported due to the job security issue [18] hence may provide a false alarm of safety because workers are less able to recognize a scaled-down capability to effectively attend a given task. Additionally, the biochemical marker sampling of fatigue like serum cortisol or blood lactate are invasive thus are not feasible to be applied for rapid, on-site FFW assessment [54]. Without the proper assessment employing valid tools, it can be tough for the worker or supervisor to predict their physiological and psychological fitness level. Studies had shown that migrant workers who made up the main construction workforce who represents a vulnerable group in terms of workplace safety [55]. The use traditional questionnaire survey alone might not be able to quantify the true fatigue level and safety related behavior due to the effect of dynamic construction environment; and workers are likely to underestimate their risk of becoming weaned off [56]. Given the deficiency and shortcoming of the subjective self-assessment survey which is less reliable [57, 58]; and the invasive biochemical test which is less practical to be applied on-site, it is recommended that the adoption of series of objective evaluation tool, in combination with the subjective scale will be a powerful approach in identifying fitness for duty capacity among the construction workers. A longitudinal day-level of fatigue or fitness indicators should be considered to examine the day-level fluctuation of energy resources which denotes fatigue and recovery [59, 60]. In the following sections, we will discuss the suitability of the objective parameters that are in parallel with the physiological and psychological change during construction works. On top of that, the compatible subjective tools to assess fatigue which have been validated specifically among the construction workers will be recommended as the complementary to the objective physical and cognitive performance metrics.

### 4.1. The physiological and psychological parameters changes related to construction work

It is disclosed that the variations in physiological and psychological parameters related to construction work pose a high risk to the workers [61]. Studies in the past attempted to employ various physiological metrics including heart rate, blood pressure, muscle activity and skin temperature to monitor real-time fatigue during the physically demanding construction tasks [37, 39, 41, 45, 62]. These parameters have the potential in providing early warning signal to detect physical strain through the continuous monitoring at work [63], especially the heart rate which has been generally considered as a reliable index of physiological strain [64]. However, the use of multiple metrics is recommended due to a higher accuracy than using a single parameter while monitoring work fatigue in construction industry [40]. In order to ease the ongoing measurement, the wearable sensing technologies developed however are subjected to technical challenges like limited validity, artifacts, lack of cutoff value for fatigue, acceptance among users and also the privacy issues, which may reduce the accuracy of such parameters in assessing real-time fatigue. To overcome these limitations, the data processing approach is important in order to minimize errors and refine the estimation of task-specific physical fatigue [63]. Other parameters related to the physical and cognitive performance measures such as static balance, muscle strength and response time have also been highlighted [37, 43]. Construction task involves manual lifting which requires several muscle groups to perform actively to attain the kinetic chain of entire body [43]. On the other hand, Postural stability was cited as the most common causes of accidents related to construction work at height. Therefore, the ability to maintain static balance is a critical factor for fall accident prevention [48].

### 4.2. Subjective evaluation tool for physical fitness

In the workplace, fatigue is a problem that is difficult to quantify, especially when looking into accidents. Moreover, no single tool serves as the gold standard for measuring fatigue due to the extensive effects of fatigue on human capacity, the challenges associated with its characterization, and its underlying causes [65]. Yet, recognizing and accurately assessing the weariness is a crucial first step towards managing it at the job.

The physical strain brought on by physical exertion during work can be measured subjectively. Borg (1970) [66] created the ratings of perceived exertion (RPE), which has been described as one of the most widely used subjective scales to evaluate whole-body and segmental strain. The perceived rating of Borg 6–20 was constructed in line with the linear relationship of the heart rate expected specific exertion level. Despite being cited as the quick and accurate way of measuring heart rates, exertion rates and work intensity in order to predict the risks for work-related musculoskeletal injuries, a single measure of RPE might not be sufficient to capture the overall range of perceptual sensations that worker experience while being physically active [67]. On the other hand, The Baecke questionnaire is a valid and reliable tool to measure the qualitative and quantitative indices addressing several dimensions of occupational physical activity, sport activities and leisure activities [68]. Although easy to apply and had been extensively used in the past few decades, this tool however subjected to recall or reporting bias due to the long recall period. Other than that, the scale is also limited to the examination of the physical aspect without addressing the cognitive dimension.

### 4.3. Subjective evaluation tool for multidimensional fitness

Workers’ self-reported fatigue symptoms and work ability in relation to work requirements, health status and the worker resources, have been reported as the most commonly used method [69, 70]. Self-reporting surveys are most frequently in field or clinical areas of occupational health [44] because they are easy to administer, less time consuming, and cost effective compared to the biomarkers and electronic device.Fatigue affects fitness for work and work performance [71, 72]. Up to date, while huge body of literature explored on the causes and consequences of occupational fatigue, limited studies have examined the fatigue assessment in the construction sector. Among the validated multidimensional fatigue evaluation scale in the construction industry were Swedish Occupational Fatigue Inventory (SOFI) [44, 46], The Fatigue Assessment Scale for Construction Workers (FASCW) [7, 8, 20], Subjective fatigue symptoms RCIF scale [35, 37]and Work Ability Index (WBI) [47, 49].

The multidimensional Swedish Occupational Fatigue Inventory (SOFI) was developed by Åhsberg et al. [73] and primarily focuses on the unique features of momentary symptom therefore are useful to examine short-termed or acute fatigue symptoms while compared to other scales which focus on chronic features of fatigue or adverse impacts resulted from the delayed recovery [74, 75]. Fatigue instruments like the multidimensional fatigue scale (MFS) and the subjective symptoms of a fatigue test (SSF) were devised to assess general populations or patients with chronic diseases, these scales however are inappropriate in evaluating instant fatigue in workers’ daily working lives [76]. The use of SOFI allows instant detection of fatigue and therefore is helpful in managing relevant safety and health issue or occupational risk in a timely manner [44]. The SOFI tool has demonstrated a satisfactory internal consistency of the subscales [5], has been translated into several languages across nations and was being tested among diverse occupational groups [77–79]. It has been recognized as the primary survey tool to measure whole-body fatigue associated with the physiological, cognitive, motor and emotional responses in which workers are able to express their feelings at the moment of study [5].

The multidimensional Fatigue Assessment Scale for Construction Workers (FASCW) was developed by Zhang et al. [32]. It consisted of only 10-items which is ease to administer taking into consideration of the literacy level of the blue-collar workers. Additionally, the tool has been studied specifically among the construction workers by [7, 20, 32, 80] and was documented its validity and reliability in the evaluation of both physical and mental fatigue in construction industry. The 10-items questionnaire consisted of 3 dimensions (physical inactiveness, mental fatigue and discomfort), with 5 possible Likert- answers where a critical score of 20 and above indicates fatigue [7]. The FASCW tool showed significant high correlations (0.66–0.71) when compared with the Fatigue subscale of the Profile of Mood States (POMS-F), indicating that the FASCW was measuring a similar construct measured by the POMS-F and had good concurrent validity. This tool also had excellent internal consistency and test-retest reliability [32].

The Finnish Institute of Occupational Health (FIOH) developed the idea of the work ability index (WAI) so that employees may assess their own ability to work based on job needs, health conditions, and mental-thinking capacities [81]. This tool decently reflects the interactions between individual physiological, mental and intellectual abilities to work, taking into considerations the working conditions, work performance capabilities, employees’ health status as well as an assessment of social characteristics [82]. Khavanin et al. had employed the WAI tool to evaluate the physical and mental fitness among the tower climbers in the construction industry, reported it as a valid and reliable scale among the manual labors working at height [49]. Another valid that had been applied in the work fatigue assessment among the high-rise construction workers such as scaffolders, steel fixers, form workers, electrician-plumbers and concreters, was the “Subjective Fatigue Symptoms RCIF Scale” defined by the Research Committee on Industrial Fatigue of Japan Society for Occupational Health, 1969. This 30-items questionnaire are classified into three domains of fatigue, namely (i) drowsiness and dullness (general fatigue); (ii) difficulty in concentration (mentally fatigue); (iii) projection of physical impairment (physical fatigue), with dichotomous answer to each fatigue symptoms [83]. Chang et al. (2009) reported those subjective fatigue symptoms highlighted in the tool were coincided with the life tyle of some workers while the extent of fatigue strains demarcated among construction workers of different task. For example, the scaffolders, steel fixers and form workers who working at height were being categorized as physically demanding fatigue task, indicated by more complaints of “projection of physical impairment” than ‘‘drowsiness and dullness” and ‘‘difficulty in concentration” post shift in comparison with pre-shift measurement [37].

Occupational fatigue research in the past decades had almost exclusively implemented subjective questionnaires assessment alone based on the self-perceived fatigue and work ability. Such subjectivity might not representative of actual human performance-based functionality thus can easily be manipulated in order to reflect the desired outcome [84]. Although subjective questionnaires are inexpensive, administering them on building sites is inconvenient and impossible. The recall bias is often reported as the biggest limitation with this strategy. The self-reported survey utilizing questionnaires, nevertheless, are unable to detect real-time physical weariness while causing little disruption to existing on-site activities [45].

### 4.4. Objective measurement of physiological metrics

Self-reported fitness might differ from the true fitness level. The development of advanced wearable sensors for real-time monitoring of physiological indices such as heart rate, skin temperature, breathing rate and electrodermal activity have provided new opportunities for the objective and continuous monitoring of physical fatigue during construction works. The sympathetic nervous system will be activated during vigorous physical activity, therefore generating specific physiological responses like the increase of heart rate, breathing rate and skin temperature. As a result, continuous monitoring these responses might be possible to identify physical fatigue besides giving clues on FFW [40, 63]. Multiple studies have employed heart rate, or heart rate in combination with breathing rate and local skin temperature monitoring to estimate physical strain among construction workers during experimental study with simulated work [35, 36, 38, 39, 42, 45, 85]. Furthermore, Aryal et al. [45] combined local skin temperature and HR measures in the development of fatigue assessment model, showed the 72% of prediction accuracy for identifying construction physical workload using both skin temperature and HR data. The wearable technologies to monitor cardiorespiratory and thermoregulatory parameters had been documented as the valid objective tool to detect physical fatigue. However the adoption of multiple, rather than single parameter is highly recommended [86]. The successful of physiological indices monitoring greatly depending on workers’ cooperation to bear with the wearable sensor while performing task, the acceptance by construction workers as well as the cost that need to be considered by the employer.

### 4.5. Objective measurement of physical performance measure

The objective work fatigue evaluation, which integrates the musculoskeletal assessment, offers detailed data on a potential employee’s physical strength and cardiovascular health to help determine whether they can perform the duties necessary for a job function [26].These performance measures will reflect a balance between work demands and the individual resources of a worker to meet those demand.

#### 4.5.1. Musculoskeletal capacity

Fatigue has been reported decrease the muscle force, strength and endurance thus reduce the ability of muscle to perform [87].The musculoskeletal capacity has been recognized as relevant individual factor to be taken into consideration as a work ability predictor in occupations with high physical demands. This element’s operational definitions include hand grip strength, balance, upper- and lower-limb endurance, trunk flexibility, and trunk flexion and extension strength [88]. The hand grip strength was defined as the predictor and adequate measurement for generalised muscle strength. Furthermore, this single useful test is low cost and suitable to be used in a time-efficient manner in construction site [35, 37, 43]. Nevertheless, the limitation of strength tests, of grip and pinch strength have been identified in which they are likely to be intentionally biased if subjects competing one another during the measurements [35]. The trunk and back endurance strength with its association with the test time, are among the important physical performance measures among the construction workers as they predict abdominal muscle functionality while the fall arrest system like harness and lanyard are worn [43].The tests were selected due to the relation to lifting and work at height capacity, most importantly easy to administer in the real work setting. Among the trunk endurance tests recommended by Mohapatra et al. including prone plank to examine trunk stability, trunk flexor endurance test, trunk extensor endurance test (as known as the Biering–Sørensen test) and trunk lateral endurance test (as known as side bridge test to evaluate the endurance of lateral core muscle). The trunk extensor test, besides assessing core endurance, has been commonly used to measure the endurance of back and hip musculature strength [43].

#### 4.5.2. Flexibility

Due to the mobility of the soft tissues surrounding the joint, physical exhaustion may alter the range of motion or flexibility of joints, leading to a condition known as fatigue-induced soft tissue shortening over time [89]. Flexibility is the capacity of a joint or group of joints to move freely and without experiencing any pain. Although everyone’s ranges of flexibility differ significantly, maintaining joint and overall body health requires certain minimal ranges. Although radiography and goniometry appear to be the finest tools for evaluating flexibility, their high technical requirements make them unsuitable for application in all contexts [90]. The sit-and-reach test, created by Wells and Dillon in 1952 and its various iterations, has historically been a part of fitness test batteries for assessing hamstring and lower back flexibility. This test is suitable for use as a flexibility measure across various populations in an occupational setting [91] including construction industry [78].

#### 4.5.3. Postural stability and control

According to the European Commission, postural stability issues are one of the most frequent reasons for accidents involving people who operate at heights [48]. Employees who work at heights must contend with a task that must be completed exactly as well as challenging external conditions, including weather like heat, humidity, high winds, and rain. Only workers with the appropriate amount of experience, qualifications, and physical and mental attributes should be given such employment [92]. The postural stability assessment was cited as the most essential element of fall accident prevention [48]. According to research linking fall risk, people control their posture by increasing the neuromuscular activity of their lower limb muscles and stiffening their ankle joints. The capacity to maintain upright posture while maintaining postural stability is thought to be a crucial component in reducing loss of balance and falls. Afferent input from the visual, proprioceptive, and vestibular systems is crucial for influencing the control of stability [93]. The one-leg standing test with eyes open (OLST-EO) and closed (OLST-EC) were frequently employed for the reliable traditional evaluation of postural stability [48]. The test is able to evaluate balance in a static position with and without a vision control. For construction workers equipped with on-site wearable inertial measurement units, Umer et al. developed a static balance monitoring tool for the proactive tracking of postural stability using a machine learning algorithm (WIMU) [39]. The created technology offers a unique and useful method to boost fall risk surveillance among construction workers carrying heavy manual material and working on slanted surfaces that might disrupt postural stability [94]. The WIMU, however, only explored a single duration of labour assignment to generate changes in static balance and only permitted the measurement of static balance rather than dynamic balance.

### 4.6. Objective measurement of cognitive performance measure

Given that FFW is multidimensional incorporating physical and mental capacity, construction tasks involve planning and thinking to exercise the cognitive function; subsequently followed by physical function execution where physical task is executed with physical strength [8]. Cognitive performance measure is therefore another important dimension of FFW to be given attention. Studies had documented the wide use of a variety of on-screen test such as Critical Flicker Fusion frequency (CFF) and simple reaction time (SRT) test to measure fatigue in healthy working population to measure the response to visual stimulus which indicates attention or concentration [95]. Other study demonstrated the prevailing indirect mental fatigue measurement by measuring the reaction time using the Personal Computer- Psychomotor Vigilance Test (PC-PVT). Independent researchers, laboratory studies, and field tests have all acknowledged PC-PVT as the only technology with solid validation evidence, and the majority of researchers believe it to be the best way to objectively evaluate fatigue [80]. The effects of acute fatigue on verbal fluency, communication, decision-making capacity, creative thinking, planning, executive control, and novelty performance have been the subject of research. Data overwhelmingly showed that even with mild degrees of weariness, all of these sophisticated cognitive tasks were severely worsened [96]. The 5-minute test was ease to be performed on construction site in the morning prior to the start of work task, and Ferrada et al. [50] reported that the mean reaction test was significantly correlated with the fatigue level indicated by self-reported sleep hours. According to the thorough analysis conducted by Dawson et al. [97], PC-PVT is the most favored objective technique for assessing fatigue at specific periods in time in the field.

### 4.7. Strength and limitations

To the best of the authors’ knowledge, this critical review is the first which provides an overview of the subjective and objective assessment tool to evaluate the fitness for construction duty in term of physical performance or cognitive performance or both, based on the existing literature. Despite being conducted using the standardized PRISMA guidelines for systematic reviews and extracting relevant studies via systematic search from three different databases, this present study has several limitations. The language bias and publication bias need to be addressed as the limitations The review process did not consider articles published in languages other than English. Moreover, the unpublished research was also not included which might potentially exclude some relevant articles. The heterogenicity or high variability among all included study in term of the working tasks, assessment tool, dimension, study population and study design did not allow the performance of meta-analysis combining the results.

The critical analysis has summarized the tools to evaluate fatigue among construction workers into two main categories, namely objective measurement instrument and the subjective self-administered survey. Construction is a dynamic industry with challenging conditions which are always evolving, which requires considerable physical and cognitive efforts. Traditional methods of recognising exhaustion rely on arbitrary questionnaires that do not enable accurate and immediate detection. These questionnaires rely on responses to predetermined inquiries about the respondent’s physical and mental states to provide a subjective assessment of weariness. Given that 20–40% of construction workers typically work above their bodies’ physiological limits and exhibit signs of weariness [58], the sensor- based physiological monitoring and computerized cognitive performance measure will be more reliable and accurate in determining the physiological state of a person, in order to detect weariness. On the other hand, the compatible subjective tools to assess fatigue which have been validated specifically among the construction workers could be recommended as the complementary to the objective physical and cognitive performance metrics.

## 5. Conclusions

This critical review provides preliminary insight on various tools and parameters used to evaluate work fatigue in the construction field, besides offering guides on the tool selection which should address the specific functional capacity required for task. Given that the construction has been recognized as a highly hazardous industry which involves huge workforce, future research should focus on the exploration of on-site practicality, the evaluation of cost benefits, and to strengthen the tool validity among construction workers of different task such as manual material handling, working and height or general worker. The findings from present review are critical for occupational health-related and human resource-related policy makers in formulating evidence-based strategies in the management of work-related fatigue and its consequences in the construction industry.

## Supporting information

S1 Checklist Preferred Reporting Items for Systematic reviews and Meta-Analyses extension for Scoping Reviews (PRISMA-ScR) checklist. (PDF)

S1 Appendix The details of mixed method appraisal tool (MMAT) assessment. (DOCX)

S1 Dataset (PDF)
