# Supplementary material for: Individual evaluation of fatigue at work to enhance the safety performance in the construction industry: A systematic review
Source: PLoS One. 2024 Feb 7;19(2):e0287892. doi: 10.1371/journal.pone.0287892 (PMC10849240; doi:10.1371/journal.pone.0287892)
Supplement: S1 Appendix — (DOCX) [file pone.0287892.s002.docx]

Appendix A. The details of mixed method appraisal tool (MMAT) assessment

| No | Author | Type of study | Score (%) | 1.1 | 1.2 | 1.3 | 1.4 | 1.5 |
| --- | --- | --- | --- | --- | --- | --- | --- | --- |
|  |  |  |  | Is the sampling strategy relevant to address the research question? | Is the sample representative of the target population? | Are the measurements appropriate? | Is the risk of non-response bias low? | Is the statistical analysis appropriate to answer the research question? |
| 1 | Hsu et al. 2008 | Quantitative | 100 | Yes | Yes | Yes | Yes | Yes |
| 2 | Li et al. 2009 | Quantitative | 60 | Yes | Can't tell | Yes | No | Yes |
| 3 | Chang et al. 2009 | Quantitative | 100 | Yes | Yes | Yes | Yes | Yes |
| 4 | Das 2014 | Quantitative | 100 | Yes | Yes | Yes | Yes | Yes |
| 5 | Wong et al. 2014 | Quantitative | 60 | Yes | Can't tell | Yes | No | Yes |
| 6 | Fang et al. 2015 | Quantitative | 80 | Yes | Can't tell | Yes | Yes | Yes |
| 7 | Zhang et al. 2015 | Quantitative | 100 | Yes | Yes | Yes | Yes | Yes |
| 8 | Zhang et al. 2015 | Quantitative | 80 | Yes | Yes | Yes | No | Yes |
| 9 | Arya et al. 2017 | Quantitative | 60 | Yes | Can't tell | Yes | Can't tell | Yes |
| 10 | Techera et al. 2017 | Quantitative | 80 | Yes | Yes | Yes | No | Yes |
| 11 | Tsai 2017 | Quantitative | 60 | Yes | Can't tell | Yes | No | Yes |
| 12 | Correia et al. 2018 | Quantitative | 80 | Yes | Can't tell | Yes | Yes | Yes |
| 13 | Cyma et al. 2018 | Quantitative | 60 | Yes | Can't tell | Yes | No | Yes |
| 14 | Khavanin et al., 2018 | Quantitative | 100 | Yes | Yes | Yes | Yes | Yes |
| 15 | Umer et al. 2018 | Quantitative | 80 | Yes | Can't tell | Yes | Yes | Yes |
| 16 | Anwer et al. 2020 | Quantitative | 80 | Yes | Can't tell | Yes | Yes | Yes |
| 17 | Galati et al. 2020 | Quantitative | 80 | Yes | Yes | Yes | No | Yes |
| 18 | Ferrada et al., 2021 | Quantitative | 100 | Yes | Yes | Yes | Yes | Yes |
| 19 | Lee et al. 2021 | Quantitative | 100 | Yes | Yes | Yes | Yes | Yes |
| 20 | Mohapatra et al. 2022 | Quantitative | 80 | Yes | Yes | Yes | Can't tell | Yes |
