# Supplementary material for: Individual evaluation of fatigue at work to enhance the safety performance in the construction industry: A systematic review
Source: PLoS One. 2024 Feb 7;19(2):e0287892. doi: 10.1371/journal.pone.0287892 (PMC10849240; doi:10.1371/journal.pone.0287892)
Supplement: S1 Dataset — (PDF) [file pone.0287892.s003.pdf]

Authors	Title	Publication	Volume	Number	Pages	Year	Publisher
Anwer, Shahnawaz; Li, Heng; Antwi-Afari, Maxwell Fordjour; Umer, Waleed; Wong, Arnold YL;	Cardiorespiratory and thermoregulatory parameters are good surrogates for measuring physical fatigue during a simulated construction task	International Journal of Environmental Research and Public Health	17	15	5418	2020	MDPI
Chang, Fu-Lin; Sun, Yih-Min; Chuang, Kao-Hsing; Hsu, Der-Jen;	Work fatigue and physiological symptoms in different occupations of high-elevation construction workers	Applied ergonomics	40	4	591-596	2009	Elsevier
Das, Banibrata;	Assessment of occupational health problems and physiological stress among the brick field workers of West Bengal, India	International journal of occupational medicine and environmental health	27		413-425	2014	Springer
Fang, Dongping; Jiang, Zhongming; Zhang, Mingzong; Wang, Han;	An experimental method to study the effect of fatigue on construction workers’ safety performance	Safety science	73		80-91	2015	Elsevier
Ferrada, Ximena; Barrios, Silvia; Masalan, Patricia; Campos-Romero, Solange; Carrillo, Juan; Molina, Yerko;	Sleep duration and fatigue in construction workers: A preliminary study	Organization, technology & management in construction: an international journal	13	1	2496-2504	2021	DeGruyter i Građevinski fakultet Sveučilišta u Zagrebu
Hsu, DJ; Sun, YM; Chuang, KH; Juang, YJ; Chang, FL;	Effect of elevation change on work fatigue and physiological symptoms for high-rise building construction workers	Safety science	46	5	833-843	2008	Elsevier

Khavanin, Ali; Malakouti, Javad; Gharibi, Vahid; Khanjani, Narges; Mokarami, Hamidreza; Ebrahimi, Mohammad Hossein;	Using Work Ability Index and work-related stress to evaluate the physical and mental fitness of Iranian telecom tower climbers	Journal of injury and violence research	10	2	105	2018	Kermanshah University of Medical Sciences
Aryal, Ashrant; Ghahramani, Ali; Becerik-Gerber, Burcin;	Monitoring fatigue in construction workers using physiological measurements	Automation in Construction	82		154-165	2017	Elsevier
Correia, Jennifer Alberti; Nonnenmacher, Letícia; Costella, Marcelo Fabiano; Pilz, Silvio Edmundo;	EVALUATION OF FATIGUE IN CONSTRUCTION WORKERS	Joint CIB W099 and TG59 International Safety, Health, and People in Construction Conference			240		
Cyma, Magdalena; Marciniak, Katarzyna; Tomczak, Maciej; Stemplewski, Rafał;	Postural stability and physical activity of workers working at height	American journal of men's health	12	4	1068-1073	2018	SAGE Publications Sage CA: Los Angeles, CA
Li, Kai Way; Yu, Rui-feng; Gao, Yang; Maikala, Rammohan V; Tsai, Hwa-Hwa;	Physiological and perceptual responses in male Chinese workers performing combined manual materials handling tasks	International Journal of Industrial Ergonomics	39	2	422-427	2009	Elsevier
Lee, Sangeun; Seong, Sojeong; Park, Soyeon; Lim, Jeeyeon; Hong, Soyun; Cho, Youngshin; Kim, Heejung;	Korean version of the Swedish Occupational Fatigue Inventory among construction workers: Cultural adaptation and psychometric evaluation	International Journal of Environmental Research and Public Health	18	8	4302	2021	MDPI
Galati, Ingrid Simone; Hermosilla, José Luís Garcia; Achcar, Jorge	Work Ability Index (WAI) and Quality of Life at Work (QLW) in the Context	Industrial Engineering and Operations Management: XXVI IJCIEOM, Rio de Janeiro, Brazil, July 8–11, 2020 26			439-449	2020	Springer

Alberto; Corvello, Flávia Motta; da Silva, Ethel Cristina Chiari;	of Occupational Accidents: A Survey with Construction Workers						
Mohapatra, Sidhiprada; Verma, Aparajita; Girish, N;	Lifting capacity prediction model using physical performance measures among construction workers	Scientific Reports	12	1	1096	2022	Nature Publishing Group UK London
Techera, Ulises; Hallowell, Matthew; Stambaugh, Nathan; Littlejohn, Ray;	Causes and consequences of occupational fatigue	Journal of occupational and environmental medicine	58	10	961-973	2016	JSTOR
Tsai, Ming-Kuan;	Applying physiological status monitoring in improving construction safety management	KSCE Journal of Civil Engineering	21	6	2061-2066	2017	Springer
Umer, Waleed; Li, Heng; Lu, Wei; Szeto, Grace Pui Yuk; Wong, Arnold YL;	Development of a tool to monitor static balance of construction workers for proactive fall safety management	Automation in Construction	94		438-448	2018	Elsevier
Wong, Del Pui-lam; Chung, Joanne Wai-yee; Chan, Albert Ping-chuen; Wong, Francis Kwan-wah; Yi, Wen;	Comparing the physiological and perceptual responses of construction workers (bar benders and bar fixers) in a hot environment	Applied ergonomics	45	6	1705-1711	2014	Elsevier
Zhang, M; Murphy, LA; Fang, D; Caban-Martinez, Alberto J;	Influence of fatigue on construction workers' physical and cognitive function	Occupational Medicine	65	3	245-250	2015	Oxford University Press UK

Zhang, Mingzong; Sparer, Emily H; Murphy, Lauren A; Dennerlein, Jack T; Fang, Dongping; Katz, Jeffrey N; Caban-Martinez, Alberto J;	Development and validation of a fatigue assessment scale for US construction workers	American journal of industrial medicine	58	2	220-228	2015	Wiley Online Library
